# Post-traumatic Middle Meningeal Artery Pseudoaneurysm Treated With Endovascular Coil Embolization

**DOI:** 10.7759/cureus.45402

**Published:** 2023-09-17

**Authors:** Ali S Yamani, Mark D Johnson, Matthew S Smith, Laura B Ngwenya, Charles J Prestigiacomo

**Affiliations:** 1 Neurosurgery, University of Cincinnati College of Medicine, Cincinnati, USA; 2 Neurology, University of Cincinnati College of Medicine, Cincinnati, USA

**Keywords:** middle meningeal artery, coil embolization, angiogram, cerebral aneurysm, pseudoaneurysm, traumatic brain injury

## Abstract

Pseudoaneurysms of the middle meningeal artery are rare events following head trauma. Given the potential for significant morbidity and mortality associated with pseudoaneurysm rupture, it is recommended that they be treated early. Endovascular embolization is a viable alternative to open surgical intervention. Here, we describe a case of an incidentally found middle meningeal artery pseudoaneurysm in a patient with a carotid-cavernous fistula after head injury. The pseudoaneurysm was treated with endovascular coil embolization.

## Introduction

Traumatic aneurysms overall are uncommon and comprise fewer than 1% of all intracranial aneurysms, with pseudoaneurysms of the middle meningeal artery (MMA) making up a small fraction of these cases [1]. Pseudoaneurysms are histologically distinct from true aneurysms with disruption of the entire arterial wall resulting in the formation of a contained hematoma outside the vessel that communicates with the artery [1,2]. Traumatic pseudoaneurysms of the MMA are associated with skull fractures of the temporal bone in 70%-90% of cases [3,4]. Notably, pseudoaneurysms can have acute or delayed presentations with a range in time to diagnosis from one day after trauma [5] to one year after the inciting event [6]. Here we describe a case of an incidentally found MMA pseudoaneurysm during the workup and treatment of a traumatic cavernous-carotid fistula (CCF) treated with endovascular coiling.

## Case presentation

**Figure 1:**
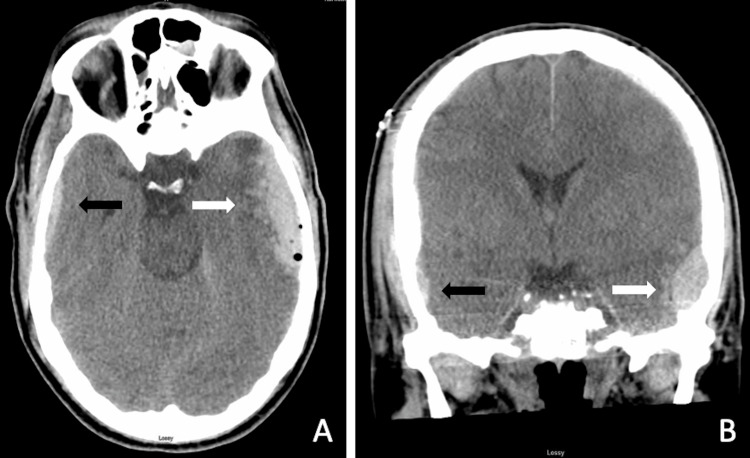
(A, B) Non-contrast CTH with left subdural hematoma and cerebral contusion (white arrow) and right epidural hematoma (black arrow). CTH, computed tomography of the head

A 59-year-old previously healthy male who was on aspirin for the primary prevention of coronary artery disease presented after being found down at home with a large laceration of the right forehead, bruising of the right eye, and abrasions on the hip and shoulder. A neurologic examination demonstrated no focal deficits. His Glasgow Coma Scale (GCS) score was 13; the patient was very confused and unable to appropriately answer questions. Initial computed tomography of the head (CTH) showed multiple facial fractures including a nondisplaced right temporoparietal fracture, left-sided extra-axial pneumocephalus suspicious for an occult left temporal fracture, bilateral cerebral convexity subdural hematomas, multifocal hemorrhagic cerebral contusions of the left frontal and temporal lobes, and a small right temporal epidural hematoma (Figures 1A, 1B). Repeat CTH at six hours showed stability of the hemorrhagic contusions and hematomas. Following a stable hospital course, the patient was discharged on hospital day 8.

**Figure 2:**
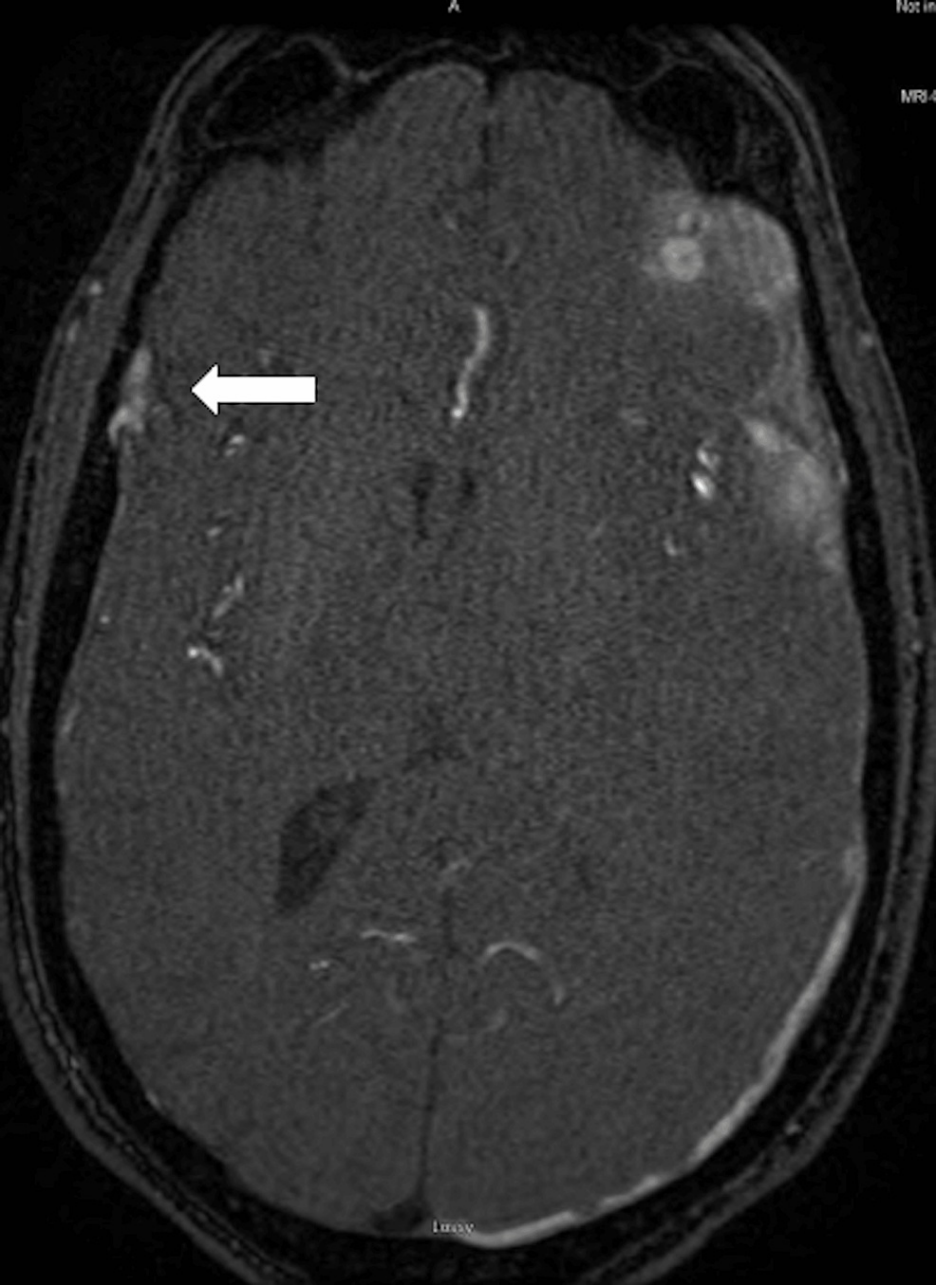
TOF MRA with high-intensity signal in the right epidural space with suspicion for active extravasation TOF MRA, time-of-flight magnetic resonance angiography

Three days following discharge, the patient re-presented with left eye chemosis, proptosis, and blurry vision. CT angiography (CTA) demonstrated bilateral enlargement of the superior ophthalmic veins suspicious for a CCF. Minimal enlargement of the right epidural collection was also noted. Magnetic resonance imaging/angiogram (MRI/A) was obtained and confirmed the presence of a left CCF, with continued enlargement of the right epidural collection, suspicious for active extravasation given the appearance of a linear hyperintensity within the collection (Figure 2).

**Figure 3:**
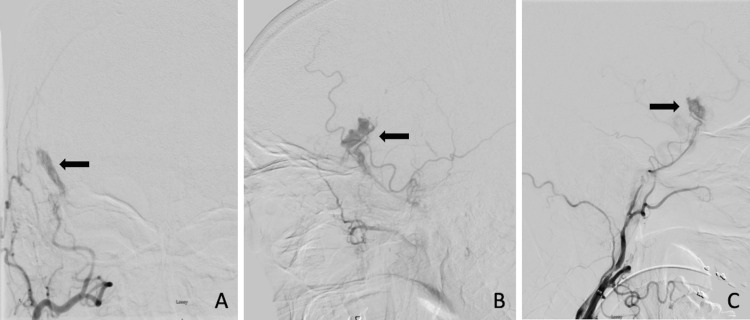
Anteroposterior (A), lateral (B), and oblique (C) views of the right ECA injection on DSA demonstrating a 1.4 x 1.6 cm pseudoaneurysm arising from the intracranial MMA ECA, external carotid artery; DSA, diagnostic cerebral angiogram; MMA, middle meningeal artery

Given the presence of the left CCF, a diagnostic cerebral angiogram (DSA) was performed to characterize the lesion. A Barrow type A fistula was confirmed on the left (not pictured) [7]. On right external carotid artery (ECA) injection, a 1.4 x 1.6 cm pseudoaneurysm arising from the MMA was noted (Figures 3A-3C). Post-DSA, the patient remained at his neurologic baseline and CTH confirmed stability of the right epidural lesion and demonstrated both subdural and epidural components of the hematoma. The epidural component was located along the right sphenoid wing and passed through the temporoparietal fracture.

**Figure 4:**
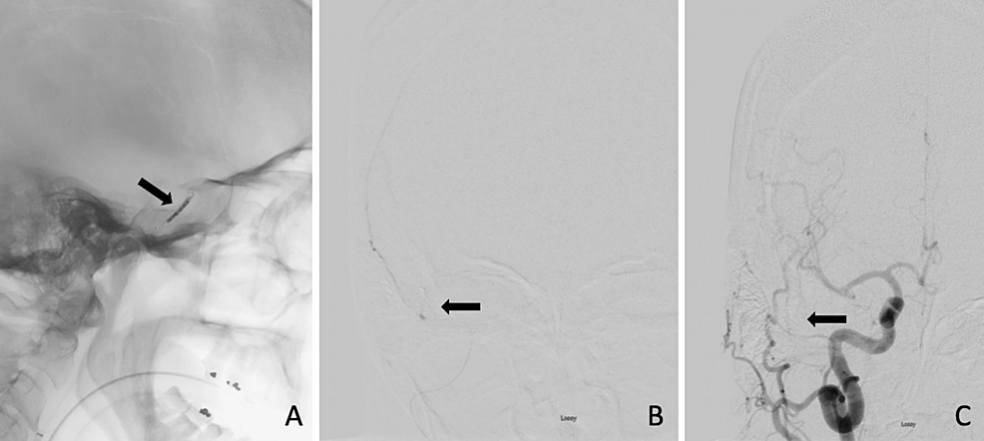
(A) Coil packing within the right MMA. (B) Post-coiling microcatheter injection of the right MMA with no residual filling of the pseudoaneurysm. (C) Three-month follow-up injection of the right CCA with stable coil mass and without filling of the pseudoaneurysm MMA, middle meningeal artery; CCA, common carotid artery

To treat the CCF, trans-arterial balloon-assisted coil embolization was recommended. Given the need for therapeutic anticoagulation with this technique, the decision was made to embolize the right MMA pseudoaneurysm prior to CCF closure. One 3D and three helical Concerto coils (Medtronic, Inc., Irvine, CA) were utilized, with good packing of the proximal MMA (Figure 4A). Microcatheter injections following coiling confirmed no residual filling of the pseudoaneurysm (Figure 4B). At the three-month follow-up, the MMA remained occluded with resolution of the pseudoaneurysm (Figure 4C). A follow-up CTH at one year from injury continued to demonstrate complete occlusion of the pseudoaneurysm.

## Discussion

Though some pseudoaneurysms may undergo spontaneous resolution without intervention [8,9], many enlarge over time and potentially rupture [10]. Resultant hemorrhage from the MMA pseudoaneurysm rupture has a reported mortality rate as high as 50% [3,4,11-13]. Given the mortality risk associated with a pseudoaneurysm rupture, early identification and treatment are imperative [10,14]. Some studies recommend obtaining a CTA in all patients with a temporal skull fracture [4,5]. Early CT findings suggestive of an MMA pseudoaneurysm include the presence of a basilar skull fracture in the temporal region, a hypodense nodule within an acute hematoma, and strong and homogenous enhancement of the hypodense nodule within an organized hematoma [15]. DSA, however, is considered the gold standard imaging modality for diagnosis and may demonstrate an elliptical or irregular aneurysmal sac without a neck, a peripheral location of the pseudoaneurysm at a distance from a branching point and delayed slow filling and emptying of contrast [10,16].

Surgical management consists of MMA ligation, coagulation, and resection [4,17]. Surgical intervention can be considered in cases with significant epidural hematoma as it allows for simultaneous evacuation, but is associated with a greater risk of intraoperative pseudoaneurysm rupture [16,18,19]. As such, endovascular embolization is considered first-line treatment of pseudoaneurysms in the absence of significant mass effect [14,19]. Modalities can include liquid embolic agents such as Onyx [12,16] or N-butyl cyanoacrylate [6,11,13] as well as endovascular coiling [2,3,5,14,17,20]. A long-term follow-up is not well characterized with regard to the risk of recurrence or recanalization, thus no specific method of embolization can be recommended at this time.

## Conclusions

Pseudoaneurysms of the MMA are uncommon but carry potential for significant morbidity and mortality. Confirmation with DSA and early treatment following diagnosis are recommended; surgical and endovascular management offers good rates of immediate occlusion. In this case, we observed an immediate complete embolization with endovascular coiling with no evidence of recurrence at one year.
